# Individuals recently abstinent from methamphetamine show selective cognitive and behavioral differences when compared to age-matched controls

**DOI:** 10.3389/fpsyt.2025.1604252

**Published:** 2025-07-11

**Authors:** M. Frances Vest, Alexandru Mihai Dumitrescu, Matthew W. Johnson, Elliot Thompson, Alfred Thomas, James C. Patterson, Kevin S. Murnane

**Affiliations:** ^1^ Department of Pharmacology, Toxicology & Neuroscience, Louisiana State University Health Science Center Shreveport, Shreveport, LA, United States; ^2^ Louisiana Addiction Research Center, Louisiana State University Health Science Center Shreveport, Shreveport, LA, United States; ^3^ Sheppard Pratt, Baltimore, MD, United States; ^4^ Department of Psychiatry and Behavioral Medicine, Louisiana State University Health Science Center Shreveport, Shreveport, LA, United States

**Keywords:** methamphetamine, response inhibition, risk taking, addiction, Cognition, delay sensitivity

## Abstract

**Introduction:**

Substance use disorders are often associated with impairments in cognitive and behavioral processes. Methamphetamine use disorder (MUD), in particular, has been linked to such differences, though it remains unclear whether response inhibition (the inability to withhold prepotent responses), risk taking, or other constructs play more prominent roles. Understanding the specific contributions of these constructs is essential for tailoring interventions and improving outcomes for individuals with MUD. This study aimed to investigate both subdomains of impulsivity in individuals recently abstinent from methamphetamine.

**Methods:**

Participants with MUD (n=29) recruited from 30-day residential treatment programs and age-matched controls (n =27) completed the Iowa Gambling Task (IGT) and Balloon Analogue Risk Task (BART) to assess risk taking and delay sensitivity, and the Stroop Color and Word Task (SCWT) and Stop Signal Task (SST) to assess response inhibition. Two-way multivariate analyses of covariance (MANCOVAs) were performed to determine group differences.

**Results:**

Analyses revealed no significant group differences in IGT net score (p=0.62) and BART average pumps (p=0.45). Conversely, significant differences emerged in as evidenced by longer stop signal reaction times (p < 0.01) and lower SCWT accuracy (p=0.03) in the MUD group compared to age-matched controls.

**Discussion:**

These findings suggest that methamphetamine use disorder is associated with specific cognitive and behavioral abnormalities. Targeting these constructs in treatment may improve outcomes for individuals recovering from MUD.

## 1 Introduction

Methamphetamine use has significantly increased in the recent years, contributing to the rise of methamphetamine use disorder (MUD) as a public health crisis (1–3). The impact of this addiction is felt not only by the individuals directly affected but also by their families and communities, with wide-ranging societal and public health implications (4–10). Beyond its societal impact, MUD is associated with neurotoxicity and systemic health deterioration, including cardiovascular, neurological and metabolic systems (1, 4, 11, 12). Importantly, MUD also leads to deficits in decision-making, risk assessment, and cognition—deficits that are central to addiction development and persistence (11, 13–17).

Key components of these cognitive deficits are constructs traditionally described as "impulsivity" (18), traits that appear pivotal in the addiction cycle. Though widely used, this term encompasses multiple, distinct cognitive processes that contribute to poor behavioral regulation. Impulsivity-related constructs include response inhibition (the ability to suppress automatic or prepotent responses), delay sensitivity (preference for immediate rewards over larger delayed ones), risk-taking behavior, and resistance to distraction or interference (19, 20). These subdomains reflect different aspects of behavioral control and decision-making, and each play unique roles in substance use disorders (18, 21, 22). Deficits in these domains may contribute to both initiation and maintenance of methamphetamine use. They can undermine self-regulation and compromise the ability to weigh long-term consequences, thereby intensifying vulnerability to relapse and diminishing quality of life (13, 23–25).

Although prior studies have separately examined these constructs in individuals with MUD (22, 26–30), few have directly compared these domains within the same population, particularly during early abstinence—a critical period for understanding relapse vulnerability. Neuroimaging studies have supported this, demonstrating alterations in fronto-striatal circuits among individuals with MUD in early abstinence, particularly in regions associated with executive function and inhibitory control (31–35). Likewise, longer lengths of abstinence have been associated with partial recovery of some of these changes in cognition and brain function (36–39). In line with prior literature, we define "early abstinence" as the period following acute withdrawal, generally within the first 7 to 30 days after last use, the period where most individuals are seeking treatment (31, 33, 34).

Of importance, there is emerging evidence that delay sensitivity and risk-taking deficits may partially recover with sustained abstinence, whereas response inhibition impairments tend to persist during early recovery (26, 39, 40). Understanding how these constructs manifests in MUD can help inform the development of targeted treatments, such as cognitive-behavioral therapies or pharmacological approaches, aimed at mitigating relapse potential. Directly comparing these constructs within the same individuals offers a more precise view of how cognitive impairments cluster and persist in MUD. Disentangling the cognitive profile of early abstinence is particularly valuable, as this period reflects a window of heightened relapse risk, but also neural recovery (22, 26, 27, 31, 36, 41–47). While these cognitive domains may share overlapping mechanisms, their divergence points can guides more targeted treatment strategies; for instance, response inhibition deficits may reflect dysfunction in fronto-striatal motor circuitry, whereas risk-related decision making may rely more heavily on dopaminergic reward pathways (21, 48–50). Characterizing which functions remain impaired during early abstinence can help guide the prioritization of treatment targets, such as whether interventions should prioritize strengthening behavioral inhibition over supporting risk-taking decision making.

To address this gap, the present study investigates different cognitive deficits in individuals recently abstinent from methamphetamine using validated behavioral tasks chosen based on their use in previous studies as it relates to MUD (22, 26–30). To assess delay sensitivity and risk taking, the Iowa Gambling Task (IGT) and the Balloon Analogue Risk Task (BART) were employed (51, 52). To assess response inhibition, the Stroop Color & Word Task (SCWT) alongside the Stop Signal Task (SST) were used (30, 53). Based on the findings of previous studies, which examined these behavioral tasks separately, we hypothesize that we will find group differences in tasks measuring response inhibition but not delay sensitivity or risk taking (22, 26–30). By concurrently examining these constructs, this study can provide a more nuanced understanding of how these deficits differentially present in a clinical setting, offering insights that may guide the development of comprehensive interventions tailored to address multiple facets of cognition.

## 2 Materials and methods

### 2.1 Participants

Recruitment took place in partnership with the Council on Alcoholism and Drug Abuse of Northwest Louisiana and the UpRising Addiction Center, two residential addiction treatment centers located in Northwest Louisiana, from June 2021 to May 2024. Participants from these treatment centers had to meet the following inclusion criteria: 1) between 25 and 55 years of age, 2) history of methamphetamine use that meets criteria for the Diagnostic and Statistical Manual of Mental Disorders 5th edition (DSM-5) criteria for a stimulant use disorder – methamphetamine subtype, 3) they have resided at the respective treatment center for at least 7 days, and are 4) English speaking. Exclusion criteria were the following: 1) meeting criteria for any other substance use disorder with the exception of nicotine and cannabis, 2) unable to sufficiently read and understand study procedures, 3) unstable medical or psychiatric conditions or disorders – including schizophrenia or type 1 bipolar disorder diagnosis clear from confusion with drug-induced states – as determined by a psychiatrist, and 4) history of significant brain injury, stroke, or seizure disorder. Out of the 149 individuals initially approached, 52 met the inclusion criteria. This group included individuals who met criteria for methamphetamine use disorder, as well as those who met criteria for both methamphetamine and cannabis use disorders. For the analysis presented here, participants with comorbid cannabis use disorder were excluded, resulting in a final sample of 29 participants diagnosed solely with MUD.

Forty-one age-matched controls were recruited from the local community from June 2021 to May 2024, which were screened based on the following inclusion criteria: 1) between 25 and 55 years of age, 2) English speaking. Exclusion criteria for age-matched controls was similar to the methamphetamine group except for 1) meet criteria for any substance use disorder except for nicotine and cannabis, with only two participants meeting criteria for cannabis use disorder. Based on these criteria and failure-to-follow-up after consenting, 27 age-matched controls were included in this analysis.

This study was approved by the Louisiana State University Health Science Center - Shreveport Institutional Review Board. All participants provided their written informed consent to participate in this study.

### 2.2 Participant characterization

#### 2.2.1 Drug use history

To capture the nature of participants' methamphetamine use, they were asked a series of questions regarding their drug use history. These questions included age of first use, duration of use, amount used per day, frequency of use, days since last use, and primary route of administration.

#### 2.2.2 Clinical screening

Participants were asked if they had received prior psychiatric diagnoses, and to indicate any diagnosis if they had. Additionally, to ensure adherence to inclusion/exclusion criteria, and clear confusion from diagnosis given during drug-induced states, individuals with methamphetamine use disorder underwent evaluation using the Quick Structured Clinical Interview for DSM-5 Disorders by a trained clinician (54).

#### 2.2.3 Medication use

Medication data were collected from all participants. In the MUD group, 58% (17/29) were taking at least one central nervous system (CNS)-active medication. These included antidepressants (n = 10), antipsychotics (n = 2), mood stabilizers (n = 5), and benzodiazepines (n = 1). In the control group, 6/27 (22%) reports any medication use, most being non-CNS-active. Of CNS-active medications, 2 participants reported antidepressant use, and 1 reported benzodiazepine use. Given the diversity and limited sample size, medication status was not included as a covariate but is described in **Table 1** and discussed as a limitation.

**Table 1:** Demographic and clinical characteristics of participants.

Variables	Methamphetamine Group (N=29)	Control Group (N=27)	p-value	F	t
Age (years), mean (SEM)	36.41 (1.03)	33.33 (1.68)	.112	4.006	1.616
				**χ* ^2^*	
Sex, n			.498	.458	
Male	16	12			
Female	13	15			
Educational Level, n			<.001	39.547	
11 Years or Less	11	0			
High School Diploma or GED	16	2			
Some College	1	9			
Associate's Degree or Tech School	1	3			
Bachelor's Degree	0	10			
Master's Degree	0	2			
Psychiatric Diagnosis, n (%)	14 (48%)	22%	.082	3.028	
n (%) Major Depressive Disorder General Anxiety Disorder Post-Traumatic Stress Disorder Attention-Deficit/Hyperactivity Disorder	9 (31%) 5 (17%) 4 (13%) 3 (10%)	3 (11%) 2 (7%) 0 (0%) 3 (11%)			
CNS-active medication, n (%)	17 (58%)	2 (7%)			
Age of First Use (years), mean (SEM)	19.56 (0.97)	–			
Duration of Abstinence (days), mean (SD) range	9.63 (3.93)7-21	–			
Duration of Use (years), mean (SEM)	13.84 (1.14)	–			
Amount Used (g/day), mean (SEM)	2.73 (0.51)	–			

### 2.3 Cognitive measures

All tasks were administered on laptop computers with a connected mouse and headphones using Inquisit 5.0 software.

#### 2.3.1 Iowa gambling task

The Iowa Gambling Task (IGT) is a well-validated measure of decision-making under uncertainty that simulates real-life situations involving reward, punishment, and learning through feedback (18). At the start of the task, participants are given a hypothetical $2000 and instructed to maximize their gains across 100 trials by selecting cards from four decks (A, B, C, and D). Each card selection leads to a gain, a loss, or a neutral outcome. Importantly, participants are not informed which decks are advantageous or disadvantageous and must learn this through trial and error over the course of the task. Two decks are considered disadvantageous, meaning they yield higher immediate rewards but are associated with larger and more frequent losses over time. In contrast, two decks are advantageous, meaning they yield smaller immediate rewards but are associated with more consistent long-term gains due to smaller losses. With this design, sensitivity to immediate reward or deficits in integrating long-term consequences into decision-making can be detected in participants who persist in selecting from disadvantageous decks. Performance is assessed by the number of advantageous and disadvantageous choices out of 100 trials as a means of measuring risk taking and delay sensitivity (i.e., advantageous choices/total trials or disadvantageous choices/total trials), as well as the net between the two (advantageous choices – disadvantageous choices) (55–57).

#### 2.3.2 Balloon analogue risk task

The Balloon Analogue Risk Task (BART) measures risk taking in another uncertain simulated context. The goal of the task is to maximize rewards over 30 trials (balloons). Participants are instructed to pump up the deflated balloon, where with each successful pump, they can earn points. Participants are faced with the choice of continuing to pump up the balloon or collect their winnings. This is contingent on successful pumps, because if the balloon pops before they collect their potential points, they lose all of their potential points for that current balloon. The main measure for this task is the average number of pumps of unexploded balloons ("adjusted pump count"), as a higher number indicates increased risk-seeking behavior (58, 59).

#### 2.3.3 Stroop color & word test

The Stroop Color & Word Test (SCWT) is a measure of response inhibition by demonstrating the interference of word meaning on the name of the color in which the words are written. This is done by measuring reaction time and accuracy differences to color-meaning congruent (e.g., the word "red" in red ink) and color-meaning incongruent (e.g., the word "red" in blue ink) combinations. Participants are given color words written in color and are asked to indicate the color of the word, not the meaning, by key pressing the coordinating key as fast as they can without making errors. Control trials for this task are colored rectangles. As per the protocol (60, 61), there are four colors (red, green, blue, and black), three color-stim congruencies (congruent, incongruent, control) and seven repetitions for a total of 84 trials.

#### 2.3.4 Stop signal task

The Stop Signal Task (SST) is a variation of a choice go/no-go task, which is another measure of response inhibition. Participants are presented with a fixation circle, which then changes to an arrow within the circle that either points left or right. When the arrow points left, participants should respond with the left arrow key, likewise when the arrow points right, participants should respond with the right arrow key. This is always the case *unless* an auditory signal is delivered after the presentation of the arrow, where participants should then stop before executing a response. They delay between presentation of the arrow (starting at 250 ms) and signal beep is adjusted up or down (50ms) depending on their performance, where a successful performance results in a longer delay (up to 1150ms) and an unsuccessful performance results in a shorter delay (down to 50 ms) (62, 63).

### 2.4 Statistical analysis

All analyses were carried out using IBM^®^ SPSS^®^ Statistics software (version 28.0.0. IBM Corporation; Armonk, NY). For all analysis, statistical significance was arbitrated at a *p* value of less than 0.05. To examine differences between the methamphetamine and control group on race, educational level and the presence of psychiatric conditions, a chi-square tests was used for these categorical variables. To examine differences between the methamphetamine and control group on age, an independent t-test was used.

Prior to conducting the main analysis, data were screened for normality using the Shapiro-Wilk test and for homogeneity of variances using Levene's test and Box's M test. The Stroop Color & Word test was the only measure to not pass tests for homogeneity of variances; therefore, multivariate effects were assessed using Pillai's Trace instead of Wilks' Lambda.

For the main analyses, a multivariate analysis of covariance (MANCOVA) was performed to examine the effect of group (control group vs methamphetamine group) on cognitive test dependent variables, while controlling for education level. The multivariate effect was assessed using Wilks' Lambda or Pillai's trace, with significance set at p < 0.05. Significant multivariate effects were followed up with univariate ANCOVAs, and effect sizes were reported using partial eta squared (η²). *Post-hoc* comparisons were conducted using Bonferroni correction to control for multiple comparisons where appropriate. For the Stop Signal Task that could not be normalized, the Mann-Whitney U test was used to compare groups on non-normally distributed variables.

## 3 Results

### 3.1 Demographic characteristics

**Table 1** details the demographic and clinical variables for the methamphetamine and control groups. The methamphetamine group had significantly lower levels of education compared to the control group. The two groups did not significantly differ on age, sex, or by the presence of a psychiatric diagnosis, which included diagnoses such as Major Depressive Disorder, General Anxiety Disorder, Post-traumatic Stress Disorder, or Attention-Deficit/Hyperactivity Disorder. Based on DSM-5 criteria for stimulant use disorder, methamphetamine subtype, participants met an average of 8.87 criteria (SD = 2.50; range = 4-11), consistent with a severe use disorder in the majority of the sample.

### 3.2 Iowa gambling task

**Figure 1:**
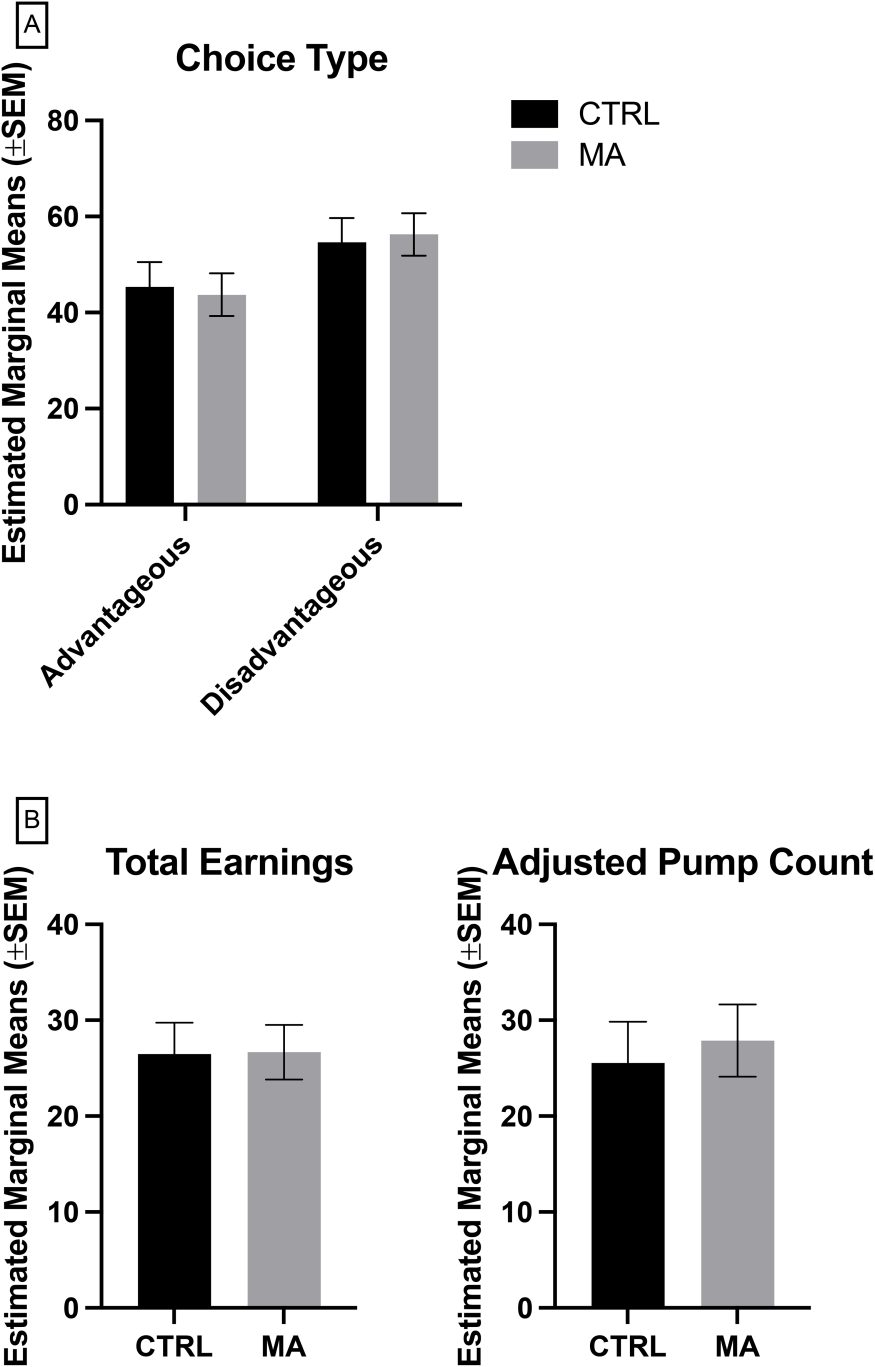
Measures of risk taking and delay sensitivity, **(A)** Iowa Gambling Task, **(B)** Balloon Analogue Risk Task, where Adjusted Pump Count is based on trials where the balloon did not pop. CTRL, control, grey (n = 27), MA, methamphetamine, black (n =29). Lack of significance determined by multivariate analysis of covariance (MANCOVA) controlling for education, followed by Bonferroni *post-hoc* for multiple comparisons.

A MANCOVA was conducted to determine the effect of group (control vs. methamphetamine) on advantageous and disadvantageous choices in the IGT, while controlling for education level. The assumptions of normality (Shapiro-Wilk test) and homogeneity of variances (Levene's test) were met. The MANCOVA revealed no significant multivariate effect of group on the combined dependent variables, Wilks' Lambda = 0.999, F (1, 50) = 0.044, p = .835, partial η² = 0.001 (**Figure 1A**). Subsequent univariate ANCOVAs indicated no significant difference between groups for advantageous choice, F(1, 50) = 0.044, p = .0835, partial η².001, and disadvantageous choice, F(1, 50) = 0.044, p = 0.835, partial η² = 0.001.

### 3.3 Balloon analogue risk task

To determine the effect of group (control vs. methamphetamine) on total earnings and adjusted average balloon count in the BART, while controlling for education level, a MANCOVA was conducted. The assumptions of normality (Shapiro-Wilk test) and homogeneity of variances (Levene's test) were met. The MANCOVA revealed no significant multivariate effect of group on the combined dependent variables, Wilks' Lambda = 0.996, F(1, 50) = 0.086, p = 0.918, partial η² = 0.004 (**Figure 1B**). Subsequent univariate ANCOVAs indicated no significant difference between groups for total earnings, F(1, 50) = 0.001, p = 0.972, partial η² < 0.001, and disadvantageous choice, F(1, 50) = 0.116, p = 0.734, partial η² = 0.002.

### 3.4 Stroop color & word test

**Figure 2:**
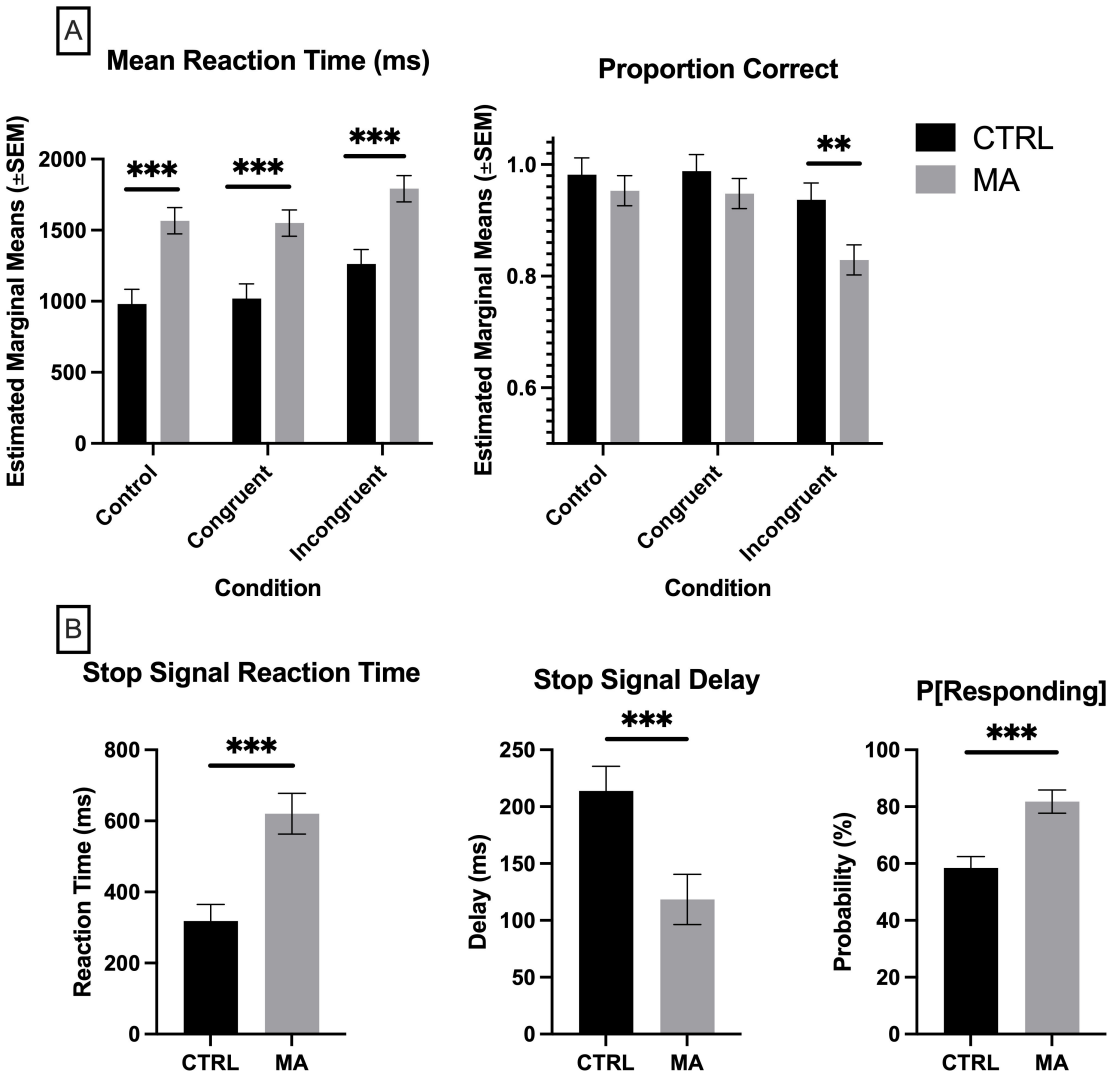
Measures of response inhibition. **(A)** Stroop Color & Word Task, **(B)** Stop Signal Task, where P[Responding], Probability of Responding on Stop Trials. CTRL, control, grey (n = 27), MA, methamphetamine, black (n =29). **(A)** Significance determined by multivariate analysis of covariance (MANCOVA) controlling for education, followed by Bonferroni *post-hoc* for multiple comparisons. **(B)** Significance determined by Mann-Whitney U test. **p <.01, ***p < 0.001.

A MANCOVA was conducted to determine the effect of group (control vs. methamphetamine) on reaction time and proportion correct in the SCWT across congruent, incongruent, and control trials, while controlling for education level. Although the assumptions of normality were met, the assumptions of homogeneity of variance were violated, as indicated by significant Levene's test and Box's M test results. Therefore, Pillai's Trace was used to evaluate the multivariate effects. The MANCOVA revealed a significant multivariate effect of group on the combined dependent variables, Pillai's Trace = 0.168, F(4, 262) = 6.024, p = <0.001, partial η² = 0.084 (**Figure 2A**). Subsequent univariate ANCOVAs indicated:

Congruent Trials: Reaction Time, F(1, 131) = 12.00, p = <0.001, partial η² = 0.084. The estimated marginal means indicated that the control group had a mean reaction time of 1018.595 ms (SE = 103.201), while the methamphetamine group had a mean reaction time of 1550.503 ms (SE = 92.738). Proportion Correct, F(1, 131) = .0397, p = 0.530, partial η² = 0.006. The estimated marginal means indicated that the control group had a proportion correct mean of 0.988 (SE = 0.030), while the methamphetamine group had a mean of 0.948 (SE = 0.027).

Incongruent Trials: Reaction Time, F(1, 131) = 11.926, p = <0.001, partial η² = 0.083. The estimated marginal means indicated that the control group had a mean reaction time of 1261.698 ms (SE = 103.201), while the methamphetamine group had a mean reaction time of 1791.951 ms (SE = 92.738). Proportion Correct, F(1, 131) = 5.714, p = 0.018, partial η² = 0.042. The estimated marginal means indicated that the control group had a proportion correct mean of 0.937 (SE = 0.030), while the methamphetamine group had a mean of 0.829 (SE = 0.027).

Control Trials: Reaction Time, F(1, 131) = 14.530, p = <0.001, partial η² = 0.100. The estimated marginal means indicated that the control group had a mean reaction time of 981.287 ms (SE = 103.201), while the methamphetamine group had a mean reaction time of 1566.585 ms (SE = 92.738). Proportion Correct, F(1, 131) = 0.397, p = 0.530, partial η² = 0.003. The estimated marginal means indicated that the control group had a proportion correct mean of 0.982 (SE = 0.030), while the methamphetamine group had a mean of 0.953 (SE = 0.027).

*Post-hoc* comparisons using Bonferroni correction revealed significant differences between groups for mean reaction time across all trial types (congruent, incongruent, and control). For proportion correct, a significant difference was found only for incongruent trials.

### 3.5 Stop signal task

A series of Mann-Whitney U tests were conducted to compare the methamphetamine group and the control group on three dependent variables from the SST: Probability of Responding on Stop Trials (P[Responding]), Stop Signal Delay (SSD), and Stop Signal Reaction Time (SSRT). Initially, the data were assessed for normality using the Shapiro-Wilk test, which indicated violations of the normality assumption for all three dependent variables. Despite attempting various data transformations (log, square root, cube square root, and inverse), the data did not meet the normality assumption. However, the data passed the assumptions of homogeneity of variance, as indicated by non-significant Levene's tests. Given the failure to achieve normality, non-parametric Mann-Whitney U tests were used to compare the groups on each variable (**Figure 2B**).

P[Responding]: The Mann-Whitney U test revealed a significant difference between the methamphetamine group (mean rank = 33.57) and the control group (mean rank = 18.25), U = 138.000, z = -3.672, p = <0.001.

SSD: The Mann-Whitney U test indicated a significant difference between the methamphetamine group (mean rank = 20.18) and the control group (mean rank = 33.88), U = 159.000, z = -3.269, p = 0.001.

SSRT: The Mann-Whitney U test showed a significant difference between the methamphetamine group (mean rank = 35.03) and the control group (mean rank = 17.29), U = 115.000, z = -4.163, p = <0.001.

## 4 Discussion

The objective of this study was to extend the literature on cognitive processes in individuals recently abstinent from methamphetamine compared to age-matched control subjects. We hypothesized that recently abstinent individuals would demonstrate significant deficits in response inhibition and not delay sensitivity or risk taking. Consistent with our hypothesis and prior studies (45, 51, 64–66), we found that our hypothesis was supported, such that there were significant differences in task performance on response inhibition tasks (SCWT & SST; **Figure 1**), but no significant differences in task performance on delay sensitivity and risk-taking tasks (IGT & BART; **Figure 2**). These findings reveal significant insight into the cognitive alterations associated with methamphetamine addiction, specifically within a population recently abstinent and vulnerable to relapse.

The observed differences in cognitive tasks, such as the SST and the SCWT, align with previous research indicating heightened response inhibition in individuals with substance use disorders (30, 65, 67, 68). Studies have consistently shown that recently abstinent individuals demonstrate impairments in response inhibition, as assessed by these tasks (27, 30, 67–71). However, caution is warranted in interpreting these findings solely as impairments in response inhibition. For instance, slower reaction times SCWT, especially in the control and congruent conditions, may also reflect generalized slowing or deficits in processing speed, which have been previously reported in stimulant-exposed populations (31, 71). Meanwhile, accuracy on the incongruent SCWT trials may better isolate interference control or response inhibition as a cognitive deficit (18). While these findings help isolate response inhibition as a specific area of concern, our results for delay sensitivity and risk-taking diverged. For example, the IGT has produced inconsistent findings across studies (14, 26, 51, 64), where IGT performance was found to be influenced by adverse childhood experiences and depression (72), or ADHD-related working memory deficits (64). In contrast, few methamphetamine studies have been published including the BART (28, 29, 51); however, of these few studies, all have shown no significant differences with upward trends towards risk-taking behavior. The IGT and BART are often used to measure decision-making, delay sensitivity and risk-taking behavior, and previous studies have reported deficits in these domains among individuals with substance use disorders such as alcohol use disorder, tobacco use disorder and general substance use disorders (45, 73–75). However, it is important to consider the role of abstinence duration in these discrepancies. Research suggests that decision-making impairments may partially recover with sustained abstinence; however, given the delineation in our findings, this recovery of cognitive function may be specific to certain domains over others (26, 31, 35, 42, 43, 76). Additionally, individual differences in the severity of addiction and comorbid psychiatric conditions could contribute to variability in task performance across studies (64, 68). These findings highlight the complexity of cognitive deficits associated with substance use disorders and underscore the need for further investigation into the factors influencing recovery trajectories in abstinent individuals.

To the best of our knowledge, this is the first study to specifically differentiate between response inhibition and delay sensitivity/risk taking deficits in individuals recovering from MUD. As this study also adds to the literature on impulsivity at different stages of recovery, the findings herein help uncover the specificity. As some literature suggests that self-report assessed trait impulsivity improves with duration of abstinence (77, 78), this study highlights the potential importance of focusing on response inhibition in recovery as a risk for relapse. While this study did not include duration of abstinence as a variable in statistical models, future studies should investigate how abstinence duration may differentially affect cognitive recovery trajectories across domains. By further understanding not just which cognitive domains are affected more but when, providers can offer tailored treatment, rather it be behavioral treatment or isolating ideal candidates for more novel therapeutic techniques such as non-invasive brain stimulation (50, 79, 80).

One limitation of the study was the significant difference of education level between the methamphetamine group and control group. To account for this, education level was used as a covariate in MANCOVA analyses. However, due to the nonparametric distribution of the SST data, a Mann-Whitney U test was used to assess group differences, which does not allow for covariate adjustment. As a result, the potential influence of education of SST performance could not be statistically controlled for, and this limitation should be considered when interpreting those findings. While medication data were collected, we were unable to statistically control for CNS-active medication use due to sample size and heterogeneity of prescriptions. Future studies should further stratify to clarify potential confounds in cognitive performance. Additionally, future studies should control for state-related variables associated with withdrawal, such as sleep, or negative affect, as these were not included in the analysis of the current study. Another limitation was that of many addiction studies, such that accounts of methamphetamine use history is all self-report, so values provided are estimates and not exact values. Biological values, such as urine drug screenings, were also not collected in this study, to assess any use of methamphetamine in our age-matched controls. Another limitation of this study is given that these participants are in a residential treatment center receiving cognitive behavioral therapy as part of their standard of care, the data indicating no deficits in the IGT should be considered with caution, as cognitive behavioral therapy has been show to improve IGT and clinician rated trait impulsivity (81, 82). Another limitation is that the IGT incorporates aspects of both delay sensitivity, risk taking, and other domains (18), allowing uncertainty which of these constructs may be involved.

The current study can act as a foundation for future research, such as studies including neuroimaging, to uncover the underlying mechanism that supports this distinction among cognitive measures. Given the direct impact methamphetamine has on the dopamine system, its influence on motor is a critical area of study. Chronic methamphetamine administration drastically depletes dopamine, so much so that it has been used as a preclinical model for Parkinson's Disease, which causes motor deficits due to dopamine neuron loss (83). Using neuroimaging modalities, the delineation of cognitive measures and how they may relate to such drastic dopamine depletion could further be explored, with preclinical studies showing distinct pathways between the two (48). In clinical populations, studies have shown mesocorticolimbic circuit-level dysfunction relating to risk taking (28) and others have shown neuroinflammation associated with severity of response inhibition (66). Similar to this study comparing the two in one population, further research could be done to explore the role of circuit-level dysfunction and neuroinflammation leading to deficits associated with these constructs. This research could be further extended with the inclusion of longitudinal studies to examine the progression of these domains from active use to acute abstinence, to long term abstinence, which would allow for more specialized treatment to be developed. Research exploring the use of interventions targeting these cognitive domains in this population would be worthwhile based on the current findings. Likewise, with larger groups, it may be unveiled that a certain level of methamphetamine use needs to be reached before seeing such delineation in impulsivity deficits.

This study found that individuals recovering from MUD exhibit response inhibition deficits but not delay sensitivity/risk taking deficits. These results suggest that response inhibition may be a more critical target for intervention in MUD recovery. While these findings are promising, they should be interpreted with caution due to limitations such as educational differences. Future research should aim to extend these findings and explore targeted interventions. With recent interest in the efficacy of noninvasive brain stimulation, some studies have explored this as a treatment option for cognitive function, with successful trials thus far (49, 50, 79, 80). Overall, this study contributes to a better understanding of cognitive function in MUD recovery and highlights the need for further research in this area.

## Data Availability

The raw data supporting the conclusions of this article are not publicly available due to ethical and legal restrictions related to participant privacy and institutional policy. De-identified data may be made available to qualified researchers upon reasonable request, contingent on approval from the study’s principal investigator and compliance with LSU Health Shreveport policies. This includes the potential establishment of a Collaborative Research Agreement or Material Transfer Agreement. Requests should be directed to Dr. Kevin Murnane (kevin.murnane@lsuhs.edu).
